# Optic Neuropathy in an Autistic Child With Vitamin A Deficiency: A Case Report and Literature Review

**DOI:** 10.7759/cureus.22074

**Published:** 2022-02-09

**Authors:** Jason Allan Seng Soon Cheah, Julieana Muhammed, Sangeetha Tharmathurai, Norhafizah Hamzah, Jamalia Rahmat

**Affiliations:** 1 Ophthalmology and Visual Science, School of Medical Sciences, Universiti Sains Malaysia, Kota Bharu, MYS; 2 Ophthalmology, Hospital Kuala Lumpur, Kuala Lumpur, MYS; 3 Ophthalmology, Hospital Tunku Azizah, Kuala Lumpur, MYS

**Keywords:** night blindness, picky eating behavior, autism spectrum disorder (asd), vitamin a deficiency, optic neuropathy

## Abstract

An eight-year-old boy with autism developed gradual onset of vision loss and nyctalopia. Dietary history revealed a diet of only French fries and potato chips for the past four years. As a result, serum vitamin A was severely below the normal level. Ophthalmologic examination revealed a normal anterior segment with bilateral optic atrophy. Vitamin A supplementation was given to restore to normal level; however, the visual impairment was irreversible. Vitamin A deficiency is common in developing countries; however, to the best of our knowledge, there are no other reported cases of permanent visual loss secondary to vitamin A deficiency in Malaysia.

## Introduction

Vitamin A deficiency (VAD) is one of the most common causes of childhood blindness in the world with around 228 million children affected [1]. It is caused by malnutrition and it may lead to ocular disease and permanent blindness. With the improvement of medical services and screening in south-east Asia, VAD prevalence has markedly reduced throughout the years. Yet, specific populations are prone to VAD, such as those with a restricted diet, abnormal metabolism, gastrointestinal malabsorption, and liver disease. In addition, VAD commonly presented with xerosis and xerophthalmia, while optic atrophy is rarely reported [1].

We would like to report a case of an autistic child with a restricted diet leading to vitamin A deficiency and visual loss. Vitamin A levels returned to normal with supplementation; however, he was left with permanent visual impairment and optic atrophy.

## Case presentation

An eight-year-old boy with underlying autism spectrum disorder (ASD) presented with gradual onset blurring of vision and loss of night vision for one month. He was noted to have difficulty in writing by the teacher in school. There was no diplopia, headache, or vomiting. His diet history revealed that he only ate french fries, potato chips, and bread, despite multiple attempts of introducing a balanced diet by the parents for the past two years. He was diagnosed as a picky eater with speech delay at the age of three years. He was born at term, with a birth weight of 3.3kg. Antenatally, the mother has gestational diabetes mellitus and was on insulin injections. He was admitted for neonatal jaundice and was on phototherapy but did not require exchange transfusion. He was the youngest among three siblings from a non-consanguineous marriage with no family history of autism. He had a history of multiple admissions for acute pharyngitis, but none for nutrition-related complications.

**Figure 1:**
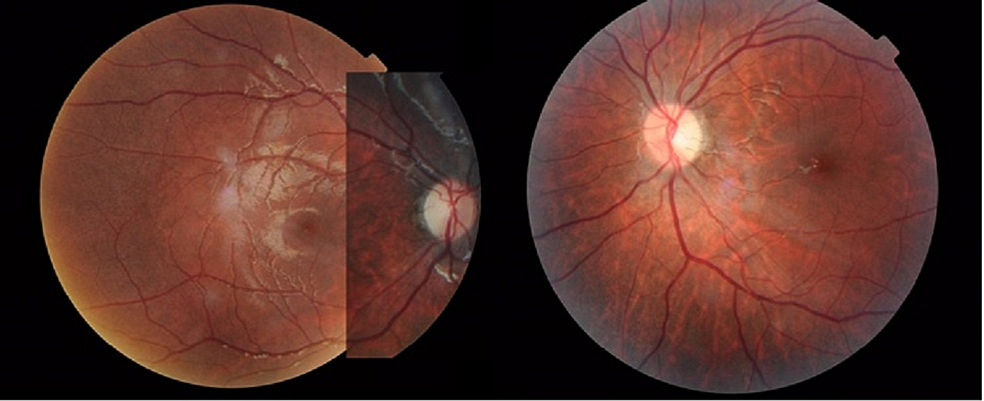
Color fundus photo of both eyes showing temporal pallor optic disc with good foveal reflex

**Figure 2:**
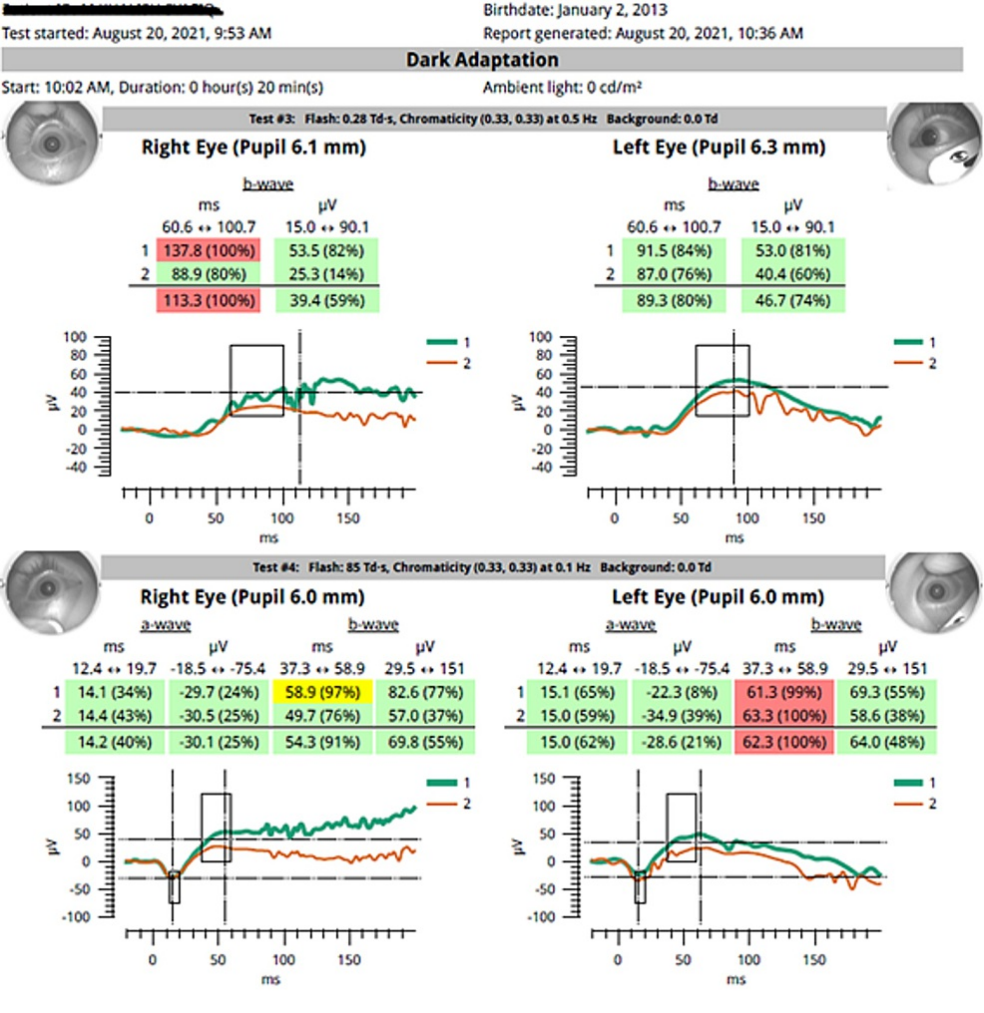
Electroretinogram showing reduced dark adaptation in both eyes

On examination, he appeared overweight with a weight of 46kg (>95th percentile), and height of 126cm (25th-50th percentile). His vision was 2/60 pinhole visual acuity (PH) same (*oculus dexter* (OD)), hand motion vision (HM) (*oculus sinister* (OS)), and no relative afferent pupillary defect was detected. The conjunctiva was white and the cornea was clear with no Bitot’s spot or xerosis of conjunctiva or cornea. Intraocular pressure of right eye was 15.5mmHg, and left eye was 17.8mmHg with iCare TA01i handheld tonometer (iCare, Revenio Group Oyj, Finland). Both eye fundus showed pallor, optic disc temporally with normal cup-to-disc ratio (CDR), and good foveal reflex (Figure 1). His blood investigation revealed severe vitamin A deficiency of 0.38 μmol/L (normal 0.9-3.0 μmol/L), vitamin B12 levels, complete blood count, and renal profile was within normal limit. The visual field on the right eye was inconclusive. Colour vision of the right eye showed a strong red-green defect. Electroretinograms were abnormal with reduced amplitude (Figure 2). Magnetic resonance imaging showed no significant abnormality. Visual evoked potential (VEP) was grossly abnormal in keeping with bilateral visual pathway defect. Diagnosis of nutritional optic atrophy secondary to Vitamin A deficiency was made.

He was started on intramuscular vitamin A 100000IU/day for three days, followed by 50000IU/ day for 14 days, and 10000/day later for two months. His serum vitamin A level returned to normal value (1.3 μmol/L) after one month of vitamin A supplementation. We followed up with him for two months and despite normalization of serum vitamin A, there was no improvement in his visual acuity. He was co-managed with pediatric team and psychiatry for eating disorders.

## Discussion

VAD is a significant public health problem worldwide that contributes significantly to the global burden of disease. VAD disorders increase the risk of infectious diseases, which can lead to increased mortality and ocular disorders such as xerophthalmia [2]. In 2009, WHO global estimates indicated that 5.2 million preschool-age children were affected with night blindness and that low serum retinol concentrations (<0.70 mol/l) affected 190 million preschool-age children [2].

Vitamin A plays a role in hematopoiesis, maintaining mucosal tissue integrity, preserving immunity response, bone growth, and ocular metabolism. Animal studies have shown that VAD causes altered bone growth. Increased osteoblastic activity as evidenced by abundant osteoid present with resultant alterations to the bones surrounding optic canals of vitamin A-deficient calves has been observed causing severe constriction and ischaemic necrosis of the optic nerve. Histological cross-section of the optic nerve with early degeneration showed areas of demyelination and fibrosis [3].

Vitamin A allows the conversion of light received at photoreceptor outer segments into electrochemical energy in the retina. It also helps in the differentiation of the stratified squamous epithelium of the ocular surface [1]. VAD causes atrophy of the normal mucosal surface with the appearance of glistening white plaques of the desquamated epithelium (Bitot’s spots), followed by wrinkling of the conjunctiva and loss of goblet cells [3].

Clinical manifestations of vitamin A deficiency include systemic and ocular signs. The systemic manifestations of VAD are hypopigmentation of hair, glossitis, anaemia, and kwashiorkor [4]. Studies done by Venkataswamy (1967) reported the most common ocular changes are night blindness (71%), followed by conjunctival wrinkling (70%), Bitot’s spot (48%), conjunctival and cornea xerosis (20%), and photophobia (12%) [5]. Other ocular changes of xerophthalmic fundus and optic atrophy are uncommon [4]. There are seven reported cases of VAD due to restricted diet in autistic children (Table 1). As shown in Table 1, the most common ocular findings are Bitot’s spots and conjunctival or cornea xerosis. However, there is only one case of irreversible visual loss reported after vitamin A level normalizes [6]. Our patient had a normal anterior segment examination with only optic atrophy leading to permanent visual loss, which is uncommon compared with cases reported. This could be attributed to the chronicity of the disease in this patient.

**Table 1:** Reported cases of vitamin A deficiency due to restricted diet in autistic children RAPD: relative afferent pupil defect

Authors	Age	Diet	Vitamin A level	Ocular manifestation
Clark et al. [7]	8	French fries	<0.35 μmol/L	Bilateral cornea punctuate epithelial erosions and mild xerosis, small corneal scar on the right eye
McAbee et al. [6]	17	Potato chips, pretzels, snack mix, cookies, and seltzer water	<0.02 μmol/L	Bilateral dry, wrinkled conjunctiva, Bitot’s spots, edema of corneal limbus, diffuse corneal haze, and bilateral optic atrophy
Chiu et al. [1]	12	Hot chips and nuggets	<0.4 μmol/L	Bilateral conjunctival and cornea keratinization, left optic atrophy
Tanoue et al. [8]	5	Fried potato, rice balls	<0.5 μmol/L	Keratinized conjunctiva, bilateral corneal ulceration, and scarring
Duignan et al. [9]	14 and 15	Bread and fried potatoes	<0.001 μmol/L	Bitot’s spot, bilateral optic disc swelling, left RAPD conjunctival and cornea xerosis
Lin et al. [10]	9	French fries only	0.1 μmol/L	Night blindness, bilateral non-healing epithelial defect, RAPD, and optic atrophy of the left eye
Steinemann et al. [11]	5	Bacon and blueberry muffin	<0.18 μmol/L	Bitot’s spot, right central cornea ulcer, conjunctiva keratinization, yellow flecks at peripheral retina

The feeding behaviour of autistic children is frequently associated with a propensity toward eating a narrow food variety, and this has been reported by many psychiatry journals [6]. In our case, the boy had a selective diet towards french fries and refused other types of food despite multiple attempts by the parents. A study done by Clark et al. reported that french fries contains less than 1% of recommended dietary allowance (RDA) of vitamin A [7]. A prolonged selective diet of french fries can lead to vitamin A and B12 deficiency.

Treatment for VAD, according to WHO guidelines, are vitamin A supplementation, intramuscular Vitamin A 100000 IU/day for three days, followed by 50000 IU/ day for 14 days and 10000 IU/day for two months in children aged eight years and above [11].

## Conclusions

VAD remains a major cause of preventable childhood blindness and mortality in developing countries. Irreversible bilateral optic neuropathies secondary to VAD are uncommon and could be attributed to the chronicity of the disease in our patient. Therefore, a high index of suspicion is needed, especially for those at risk of nutritional optic neuropathy. Early screening in high-risk groups will allow early intervention. This may reduce the risk of permanent visual loss in patients with VAD.
